# Localization and Pixel-Confidence Network for Surface Defect Segmentation

**DOI:** 10.3390/s25154548

**Published:** 2025-07-23

**Authors:** Yueyou Wang, Zixuan Xu, Li Mei, Ruiqing Guo, Jing Zhang, Tingbo Zhang, Hongqi Liu

**Affiliations:** 1 Aerospace Research Institute of Materials and Processing Technology, Beijing 100076, China; wangyueyougood@163.com (Y.W.);; 2 Huazhong School of Mechanical Science and Engineering, University of Science and Technology, 1037 Luoyu Road, Wuhan 430074, China; m202270656@hust.edu.cn

**Keywords:** surface defect segmentation, deep learning, machine vision, two-stage model

## Abstract

Surface defect segmentation based on deep learning has been widely applied in industrial inspection. However, two major challenges persist in specific application scenarios: first, the imbalanced area distribution between defects and the background leads to degraded segmentation performance; second, fine gaps within defects are prone to over-segmentation. To address these issues, this study proposes a two-stage image segmentation network that integrates a Defect Localization Module and a Pixel Confidence Module. In the first stage, the Defect Localization Module performs a coarse localization of defect regions and embeds the resulting feature vectors into the backbone of the second stage. In the second stage, the Pixel Confidence Module captures the probabilistic distribution of neighboring pixels, thereby refining the initial predictions. Experimental results demonstrate that the improved network achieves gains of 1.58%±0.80% in mPA, 1.35%±0.77% in mIoU on the self-built Carbon Fabric Defect Dataset and 2.66%±1.12% in mPA, 1.44%±0.79% in mIoU on the public Magnetic Tile Defect Dataset compared to the other network. These enhancements translate to more reliable automated quality assurance in industrial production environments.

## 1. Introduction

In industrial manufacturing, product surfaces often exhibit various types of defects due to process fluctuations and environmental disturbances [1,2]. When the defect area exceeds a critical threshold, the mechanical properties and surface quality of the product are significantly compromised. Therefore, accurate defect area estimation is essential for quality control.

As a fundamental step in defect area estimation, the precision of defect segmentation algorithms directly affects the accuracy of area computation. Compared to traditional methods that rely on manual annotation, automated segmentation based on machine vision has emerged as a key research focus in intelligent manufacturing, owing to its efficiency, accuracy, and objectivity [3,4,5]. Existing machine vision approaches can be broadly classified into two categories: traditional methods and deep learning-based methods. Traditional approaches extract defects by manually defining feature detection rules [6,7,8]. However, due to the complex textures of surfaces and the diversity of defect types, these methods require the frequent adjustment of feature parameters across different conditions, limiting their generalization capability [3,9]. In contrast, deep learning methods can automatically learn multi-scale defect features from input data, reducing their reliance on prior knowledge. As a result, they have been widely adopted in industrial applications [10,11].

**Figure 1:**
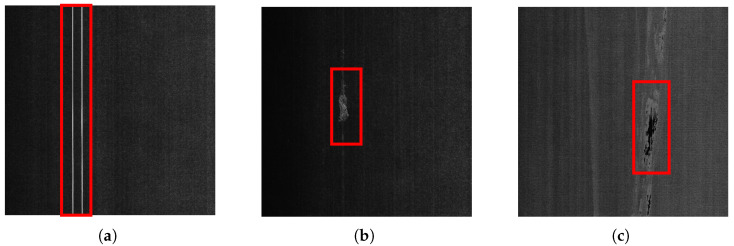
Imbalanced foreground–background area distribution across different defect types. (**a**) Gap defect. (**b**) Hairball defect. (**c**) Lack of coating defect.

**Figure 2:**
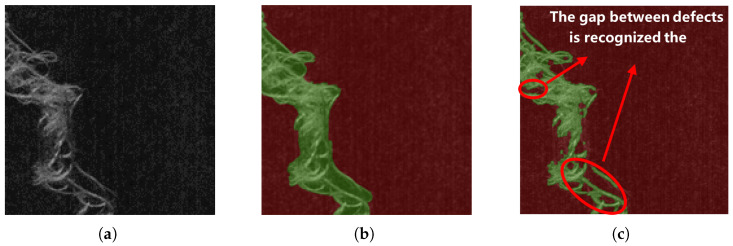
Over-segmentation of internal gap regions within defects. (**a**) Original image. (**b**) Ideal segmentation result. (**c**) Common algorithm segmentation results.

However, in specific application scenarios, such as fabric surface defect detection and magnetic tile inspection, deep learning-based segmentation algorithms still face two major technical challenges [12,13,14]:

- As shown in Figure 1, the background region occupies a significantly larger area than the defect region. This imbalance causes small defects to be overlooked during training, leading to a degraded segmentation performance. Although existing studies have attempted to address this issue by improving sample-selection strategies, designing class-sensitive evaluation metrics, and developing weighted loss functions, several problems remain. These include limited model convergence efficiency, high sensitivity to hyperparameters, and poor cross-domain generalization [15,16].
- As shown in Figure 2, there are numerous gap areas within the specific type of defect, and these gap areas are located above the background area. When relying solely on pixel value, texture, or morphological features for region partitioning [17,18], these internal gaps are often misclassified as being part of the background. This over-segmentation results in underestimated defect areas compared to the ground truth.

To address these challenges, this study proposes a two-stage segmentation network named the Localization and Pixel-Confidence Network (LPC-Net), which integrates a Defect Localization Module (DLM) and a Pixel Confidence Module (PCM). Specifically, the DLM is designed to mitigate the issue of area imbalance, while the PCM is dedicated to alleviating the over-segmentation of internal gaps.

The main contributions of this paper are summarized as follows:

(1) LPC-Net is proposed. It addresses two key challenges in surface defect segmentation: foreground–background area imbalance, and the over-segmentation of internal defect gaps. Experimental results show that LPC-Net improves mean Pixel Accuracy (mPA) and mean Intersection over Union (mIoU) compared to baseline and state-of-the-art (SOTA) methods.
(2) A DLM Module is introduced. It estimates the location of defects and embeds the spatial information into the loss function of the second-stage backbone through weighted coefficients. By dynamically adjusting pixel-wise loss weights, the model’s sensitivity to defect regions is enhanced, mitigating the adverse effects of area imbalance.
(3) A PCM is introduced. It captures the probabilistic distribution of neighboring pixels from the initial segmentation results and generates pixel confidence matrices to guide the model’s decision-making. It reduces the misclassification of fine internal gaps within defects.

The rest of the paper is structured as follows: Section 2 reviews related works on the methods of image segmentation, feature enhancement, and handling area imbalance and discusses the limitations of existing approaches. Section 3 describes the overall structure of LPC-Net, including the design and functionality of the PCM and DLM, as well as the loss functions used in training. Section 4 presents experimental results on both a self-built dataset and a public dataset, and conducts ablation studies to verify the effectiveness of the PCM and DLM. Section 5 concludes this paper.

## 2. Related Works

### 2.1. Image Segmentation

Image segmentation methods can be broadly categorized into traditional approaches and deep learning-based approaches. Traditional methods typically divide an image into non-overlapping regions based on differences in greyscale, texture, or shape features. Representative techniques include thresholding, region growing, watershed algorithms, clustering-based methods, conditional random fields, and graph cuts. These methods offer good segmentation accuracy and speed in specific scenarios. However, they often require manually designed feature extractors tailored to specific scenarios. This limits their adaptability in complex and dynamic industrial environments.

With the development of deep learning, a range of deep learning-based segmentation models has emerged. These models demonstrate strong segmentation capability and good generalization. They often achieve high accuracy on public datasets [10]. Based on architectural differences, these models can be roughly classified into several categories: fully convolutional networks (FCNs) [19], encoder–decoder architectures [20,21], multi-scale feature pyramid networks [22], R-CNN-based models [23], and dilated convolution structures [24].

Fully convolutional networks replace fully connected layers with convolutional layers, enabling the network to process inputs of arbitrary size. Ji et al. [25] proposed a parallel FCN that integrates holistically nested edge detection (HED) to capture edge information. Encoder–decoder networks extract features with the encoder and perform pixel-level segmentation with the decoder. Zhang et al. [26] designed a novel encoder–decoder framework combining a Swin Transformer and a CNN-based decoder for high-resolution remote sensing image segmentation. Feature pyramid networks enhance segmentation by leveraging multi-scale features. Hu et al. [27] proposed a multi-scale CNN that fuses intermediate feature maps to capture diverse representations. R-CNN-based models combine region proposals with deep convolutional networks. They use selective search to identify candidate regions, extract features, and generate segmentation masks. Liu et al. [28] developed a region-based CNN that performs pixel-level segmentation of fine cracks on asphalt surfaces. Dilated convolution structures expand the receptive field without increasing computational cost. Xie et al. [29] proposed a context hierarchical integrated network (CHI-Net) that uses four cascaded branches of dense dilated convolutions to capture multi-scale features.

With the ongoing innovation around network modules and the emergence of diverse combinations, deep learning-based segmentation techniques are increasingly adopted in industrial applications. However, general-purpose models often require adaptation to the specific properties of defects in real-world scenarios to achieve optimal performance.

### 2.2. Feature Enhancement

As a key step in image segmentation, feature enhancement can effectively improve segmentation quality when the image resolution is low or features are indistinct. Traditional enhancement techniques include the following: contrast enhancement, edge enhancement, noise suppression, and multi-scale analysis.

In recent years, many researchers have integrated feature enhancement strategies into deep learning networks for segmentation tasks. Wang et al. [30] proposed a contrast-enhancement preprocessing method that mitigates the object blurring or overexposure caused by lighting, thereby improving segmentation accuracy. Li et al. [17] designed an edge-detection-based enhancement module to fuse edge and spatial features. Li et al. [18] introduced a Reflection Suppression Block (RSB) using Laplacian convolution to extract edge features more effectively, reducing the impact of surface reflection noise on segmentation accuracy. Dai et al. [31] developed a Dual-Branch Multiscale Aggregation (DBMSA) module to capture deep multiscale semantic features and optimize object boundaries through multi-stage feature fusion.

However, these methods primarily guide segmentation based on greyscale, texture, and shape features. They do not explicitly incorporate the probability distribution of neighborhood pixels as auxiliary information in the network. As a result, fine gaps within defects are often misclassified as background due to over-segmentation.

### 2.3. Area Imbalance

An imbalanced area distribution between defect and normal regions is a common issue in image segmentation. This is particularly evident in industrial image processing, where defect regions often occupy much smaller areas compared to normal regions. To address this challenge, researchers have proposed various strategies to improve model sensitivity and accuracy under limited-sample conditions. These include loss function reconstruction, hard negative mining, region proposal networks, and foreground data augmentation.

Lin et al. [14] introduced an improved cross-entropy loss function, Focal Loss, by reducing the loss weight of well-classified samples, enabling the network to focus on hard samples. R. G. et al. [23] applied a bootstrap strategy, starting with a small subset of training data to initialize the model. Misclassified negative samples were then collected to form a hard negative set for further training and iterative model refinement until convergence. Ren et al. [32] proposed a Region Proposal Network (RPN), which detects potential object regions and their boundaries to generate high-quality proposals. These proposals guide the backbone network to focus on high-confidence areas. Zhang et al. [33] presented ObjectAug, an object-level augmentation method, which separates objects and backgrounds using ground-truth labels, applies data augmentation to the object, restores artifacts using image in-painting, and recombines the object and background into an enhanced image.

However, these methods have limitations. Reconstructed loss functions are sensitive to hyperparameter settings. Hard negative mining is time-consuming and difficult to integrate into end-to-end models. Region Proposal Networks face performance bottlenecks when processing high-resolution images or large-scale datasets. Foreground data augmentation can lead to overfitting on specific features of training samples, reducing its generalization to unseen data.

## 3. Methods

### 3.1. Overall Network Architecture

Deeplabv3+ has been widely adopted for image segmentation tasks due to its unique encoder–decoder architecture with Spatial Pyramid Pooling (SPP) [34]. In this study, Deeplabv3+ is selected as the backbone network for surface defect segmentation. The backbone consists of multi-parameter convolutional layers (including the convolution operation, batch normalization, and activation function layers), max-pooling layers, bottleneck modules, Atrous Spatial Pyramid Pooling (ASPP) modules, and upsampling layers. Specifically, the bottleneck modules extract features at multiple depths. The ASPP module captures features at different receptive fields using multi-scale atrous convolutions. The upsampling layers apply bilinear interpolation to achieve pixel-level segmentation.

To address the challenges of foreground–background area imbalance and the over-segmentation of fine internal gaps, two modules are integrated into the backbone: the DLM and the PCM. The DLM is a lightweight neural network designed to estimate the approximate location of defects. The PCM consists of a decision module, manually configured convolutional layers, and an averaging matrix. It is responsible for refining the segmentation results.

**Figure 3:**
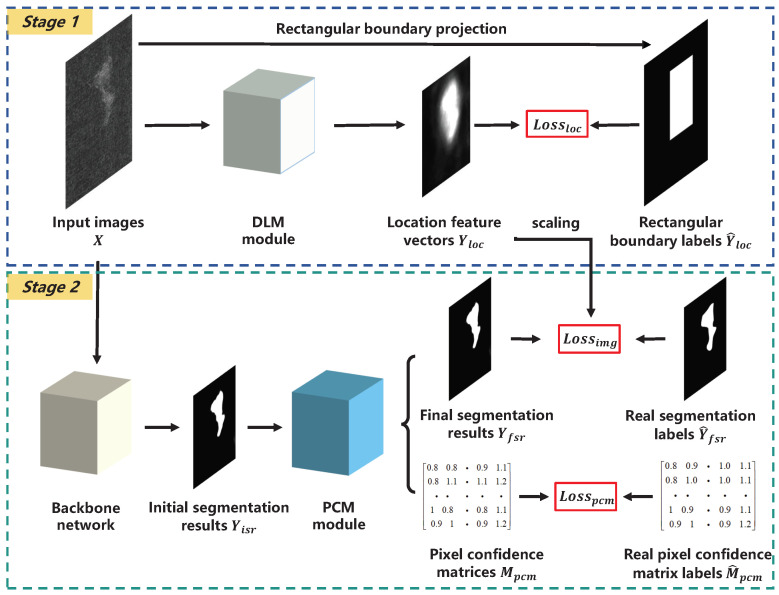
The process of LPC-Net.

**Figure 4:**
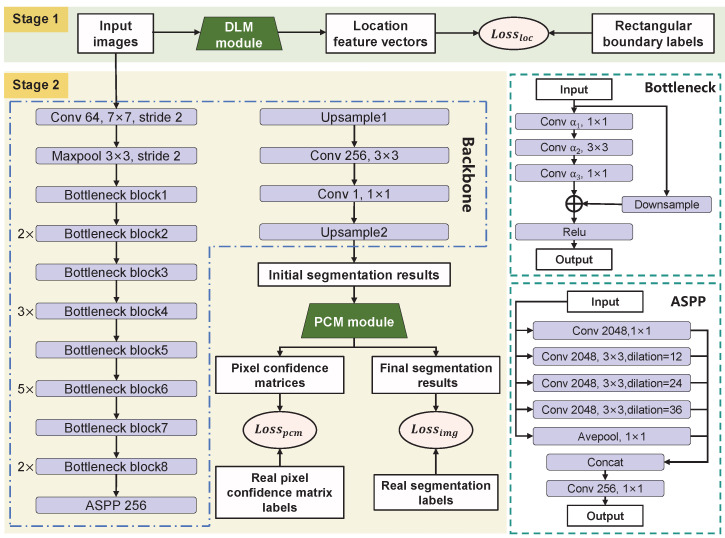
The detailed structure of LPC-Net.

As illustrated in Figure 3, the training process consists of two stages. In the first stage, the DLM takes input images *X* and generates location feature vectors $Y_{loc}$. These are supervised using automatically generated rectangular boundary labels ${\hat{Y}}_{loc}$ to perform the coarse localization of defect regions. The image loss function $Loss_{img}$ is dynamically adjusted by assigning different weights to each pixel, enhancing the model’s sensitivity to defect features. In the second stage, the PCM calculates the probabilistic distribution of neighboring pixels based on the initial segmentation results $Y_{isr}$. It generates spatially correlated pixel confidence matrices $M_{pcm}$, which are used to refine $Y_{isr}$, producing the final segmentation results $Y_{fsr}$. The overall network architecture is illustrated in Figure 4, and detailed parameters of the backbone layers are listed in Table 1.

### 3.2. Defect Localization Module

Lin’s study shows that extreme foreground–background class imbalance during training can degrade a network performance [14]. Inspired by the two-stage architecture of R-CNN, this study introduces a lightweight neural network group to roughly locate defect regions without significantly increasing computational cost. This localization serves as guidance for the subsequent backbone network, thereby mitigating the adverse effects of class imbalance.

**Figure 5:**
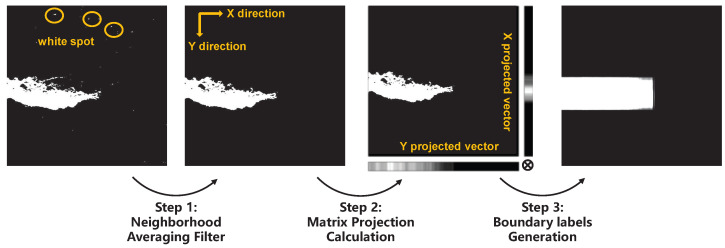
The process of obtaining the rectangular boundary labels.

The training process begins by generating rectangular boundary labels ${\hat{Y}}_{loc}$ using the rectangular boundary projection method, and then serves ${\hat{Y}}_{loc}$ as the ground truth for the DLM. The construction steps are illustrated in Figure 5. First, for each pixel in the original image, the average pixel value within a 10×10 neighborhood is calculated, and pixels with an average value below 0.2 are set to 0. Next, based on matrix projection principles, pixel value projection vectors are computed along both the X-axis and Y-axis. Finally, the outer product of the X and Y projection vectors is calculated to obtain the final ${\hat{Y}}_{loc}$. It should be specifically noted that the first step is designed to filter out small white spots in the background to prevent noise from interfering with boundary construction.

**Figure 6:**
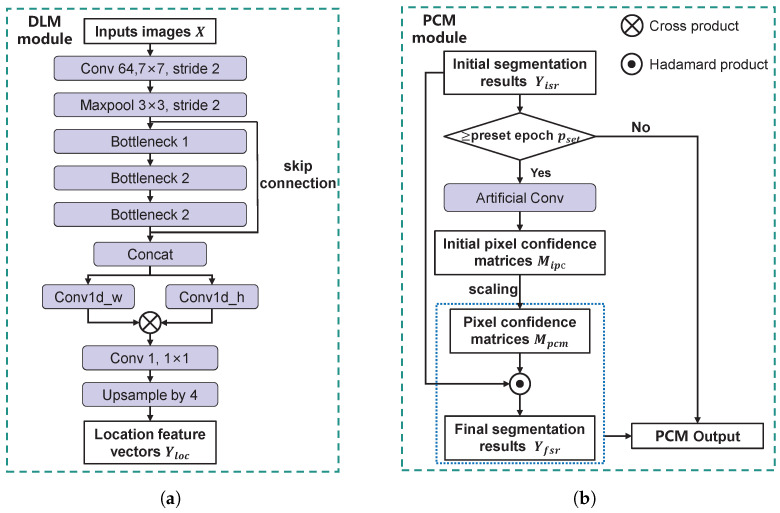
The detailed structures of the DLM and PCM. (**a**) The DLM. (**b**) The PCM.

The architecture of the DLM is shown in Figure 6a. It consists of convolutional layers, three bottleneck modules, a row convolution layer, a column convolution layer, and an upsampling layer. Let the input image be $X\in R^{n\times C\times H\times W}$, where *H* and *W* denote the height and width, *C* is the number of channels, and *n* represents the number of samples. The processing flow is as follows: First, shallow features are extracted from *X* via convolution and max-pooling layers. Then, deep features are obtained using three bottleneck modules. A skip connection fuses shallow and deep features, resulting in $\bar{X}\in R^{n\times C^{\prime }\times H/4\times W/4}$, where $C^{\prime }$ denotes the total number of channels after fusion. Next, $\bar{X}$ is separately passed through the row and column convolution layers to produce row feature vectors $\;{\bar{X}}_{w}\in R^{n\times C^{\prime }\times 1\times W/4}$ and column feature vectors ${\bar{X}}_{h}\in R^{n\times C^{\prime }\times H/4\times 1}$. The two dimensions of ${\bar{X}}_{w}$ and ${\bar{X}}_{h}$ are multiplied to obtain the reconstructed feature map $\tilde{X}\in R^{n\times C^{\prime }\times H/4\times W/4}$. Finally, a 1×1 convolution is applied to compress the channel number to *C*, and the result is upsampled to produce the location feature vectors $Y_{loc}\in R^{n\times C\times H\times W}$. The purpose of introducing row and column convolutions is to mimic the generation process of the rectangular boundary label, thus increasing the network’s fitting capability.

To enable the output of the DLM to guide the second-stage network training, a linear mapping is applied to $Y_{loc}$ to generate the corresponding compensation matrices $M_{comp}$, as defined below:

(1) $$M_{comp}=1+\omega \cdot {Norm}_{-1-1}\left(Y_{loc}\right)$$

where ω denotes the compensation coefficient and ${Norm}_{-1-1}$ indicates normalization to the range [−1, 1]. Each element (i,j) in $M_{comp}$ represents the probability that the corresponding pixel belongs to a defect region. A larger value indicates a higher likelihood of the pixel being part of a defect. When computing the image loss function ${Loss}_{img}$ in the second-stage backbone, higher loss weights should be assigned to such pixels.

**Figure 7:**
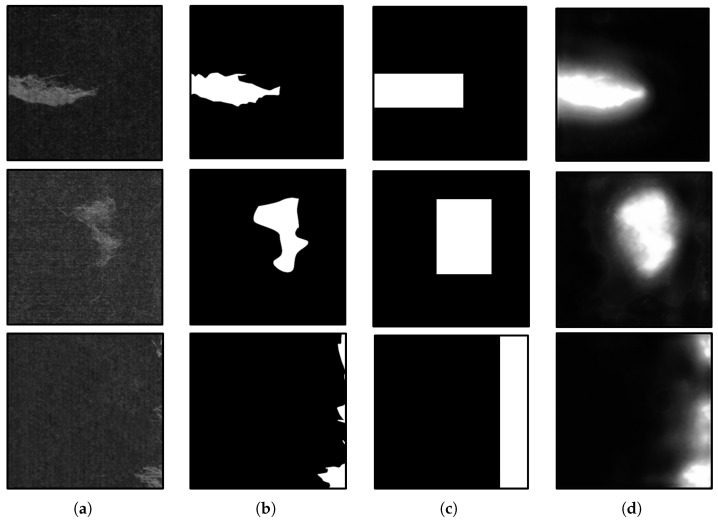
The detailed structures of the DLM and PCM. (**a**) Original images. (**b**) Ground truth. (**c**) Rectangular boundary labels. (**d**) Location feature vectors.

Figure 7 illustrates the preliminary localization results of the DLM. As shown in the column (d) output, the DLM effectively localizes defect positions in the input samples. The closer a pixel is to the center of a defect, the higher the corresponding value in $Y_{loc}$.

Essentially, this method enhances the penalty for the discrepancy between the segmentation results and the true labels, thereby mitigating the foreground–background area imbalance. Unlike conventional two-stage segmentation networks, the proposed approach benefits from the automatic generation of ${\hat{Y}}_{loc}$ by the DLM without requiring manual annotation. Although replacing precise defect contours with rectangular regions in $\hat{Y}loc$ may introduce noisy annotations, this is a deliberate design choice: the DLM only provides preliminary localization rather than precise boundary delineation. Critically, as experimentally validated in Section 4.4, this coarse localization has a minimal detrimental impact on the final segmentation accuracy of the second stage. Furthermore, this design confers significant practical advantages: precisely because the DLM’s task is simplified to approximate localization, a highly lightweight network can be employed. This ensures computational efficiency and maintains runtime within strict industrial constraints.

### 3.3. Pixel Confidence Module

The PCM is designed to address the over-segmentation of fine internal gaps within defect regions by leveraging the statistical distribution of neighboring pixel probabilities. The assumption is as follows: if all surrounding pixels of a given pixel are classified as a defect, the likelihood of that pixel belonging to the defect region is high. Conversely, if the surrounding pixels are all background, the pixel is likely to be the background. Based on this principle, the PCM refines the initial segmentation by analyzing the local probability distribution, thereby reducing false negatives in small defect gaps, particularly when these gaps are narrow.

**Figure 8:**
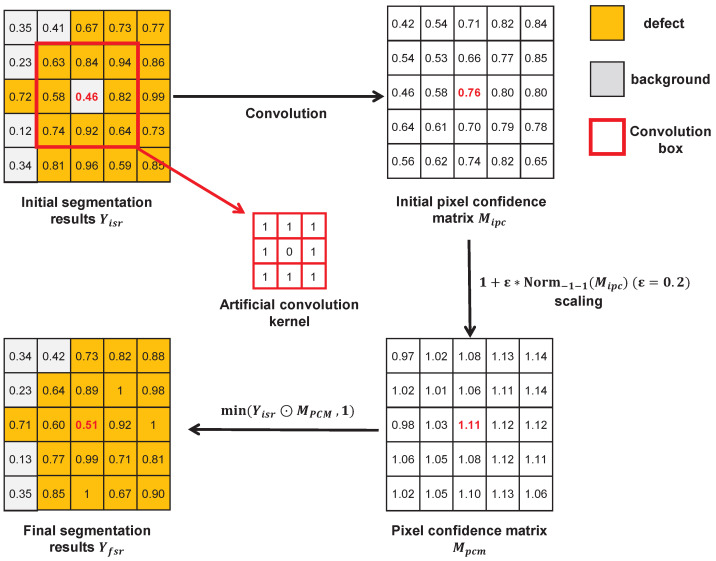
The process of the PCM module correcting pixels.

Figure 8 illustrates the operational mechanism of the PCM. First, the initial segmentation results $Y_{isr}$ are convolved with a fixed convolution kernel to obtain the initial pixel confidence matrices $M_{ipc}$. These matrices are then linearly scaled to obtain pixel confidence matrices $M_{pcm}$ whose elements range between 1±ε. The final segmentation results $Y_{fsr}$ are obtained by taking the element-wise product of $Y_{isr}$ and $M_{pcm}$, and then taking the element-wise minimum between each resulting element and 1. Comparing the highlighted element (red element) in the $Y_{isr}$ and $Y_{fsr}$ in Figure 8, the element in $Y_{isr}$ is initially classified as the background (assuming a threshold of 0.5), and then it is reclassified as a defect in $Y_{fsr}$ after correction by $M_{pcm}$. In practice, the kernel size, parameters, and ε can be adjusted based on task-specific needs. The kernel weights remain fixed throughout the training process.

However, directly applying the PCM correction described above introduces two potential issues:

1. Increased misclassification at defect boundaries: In edge regions, especially where boundary kurtosis is high (e.g., sharp tips or needle-like protrusions), the PCM may incorrectly assign background labels to true defect pixels. This occurs because the surrounding pixels are predominantly background, lowering the confidence score of the edge pixel after PCM processing.
2. Parameter explosion: Even with small ε, prolonged training can lead to value saturation in $Y_{isr}$ and $M_{pcm}$, pushing them toward binary extremes (0 or 1), compromising segmentation accuracy.

To mitigate these issues, this study introduces two countermeasures. For Issue 1, experimental results indicate that applying the PCM module during the early stages of backbone training increases the difficulty of defect boundary segmentation. Therefore, a predefined iteration threshold $p_{set}$ is introduced. The PCM is only activated once the training iteration exceeds $p_{set}$, ensuring that the backbone has already learned relatively stable and smooth boundaries. This reduces the negative impact of PCM corrections at the edges. For Issue 2, a pixel confidence matrix loss function ${Loss}_{pcm}$ is added during training. Under the joint constraint of ${Loss}_{img}$ and ${Loss}_{pcm}$, both $Y_{isr}$ and $M_{pcm}$ progressively converge toward their respective ground truth ${\hat{Y}}_{isr}$ and ${\hat{M}}_{pcm}$, thereby preventing parameter explosion.

As illustrated in Figure 6b, PCM implementation proceeds as follows: the module receives $Y_{isr}$ from the backbone and checks whether the current training epoch exceeds $p_{set}$. If not, PCM bypasses all operations and outputs $Y_{isr}$ unchanged. Once the threshold is surpassed, $Y_{isr}$ is convolved with a fixed kernel to generate $M_{ipc}$, which is then linearly mapped to obtain $M_{pcm}$. The final segmentation output $Y_{fsr}$ is computed via element-wise multiplication of $Y_{isr}$ and $M_{pcm}$. At this stage, the PCM outputs both $Y_{fsr}$ and $M_{pcm}$. During training, the ground truth ${\hat{Y}}_{fsr}$ is manually annotated, while ${\hat{M}}_{pcm}$ is obtained by convolving ${\hat{Y}}_{fsr}$ with a kernel of identical size and parameters.

### 3.4. Loss Function

The LPC-Net involves three types of loss functions: defect localization loss ${Loss}_{loc}$, image loss ${Loss}_{img}$, and pixel confidence matrix loss ${Loss}_{pcm}$. ${Loss}_{loc}$ is applied during the first training stage to evaluate the discrepancy between $Y_{loc}$ and ${\hat{Y}}_{loc}$. ${Loss}_{img}$ is used in the second stage to measure the difference between $Y_{fsr}$ and ${\hat{Y}}_{fsr}$. ${Loss}_{pcm}$ is also applied in the second stage and quantifies the deviation between $M_{pcm}$ and ${\hat{M}}_{pcm}$. Unlike ${Loss}_{img}$, ${Loss}_{pcm}$ is only incorporated into the total loss after the second-stage training surpasses $p_{set}$.

All three loss functions adopt the Binary Cross Entropy (BCE) loss to measure the similarity between the predicted and true labels, defined as follows:

(2) $$BCE\left(Y,\hat{Y}\right)=-\frac{1}{N}\sum_{i=1}^{N}\left[{\hat{Y}}_{i}log\left(Y_{i}\right)+\left(1-{\hat{Y}}_{i}\right)log\left(1-Y_{i}\right)\right]$$

where *N* denotes the number of samples, $Y_{i}$ represents the predicted value, and ${\hat{Y}}_{i}$ is the corresponding ground truth for the *i*-th sample. It is important to note that, for ${Loss}_{img}$, the contribution of each pixel to the overall loss is weighted unequally. These weights are dynamically adjusted by the $M_{comp}$ generated by the DLM.

The overall loss function for LPC-Net is defined as follows:

(3) $$Loss_{total}=\left\{\begin{array}{cc} Loss_{loc} & ,\mathrm{stage}1\\ Loss_{img} & ,\mathrm{stage}2,\;\mathrm{currenct}\;\mathrm{epoch}\;\leq p_{set}\\ \lambda_{1}Loss_{img}+\lambda_{2}Loss_{pcm} & ,\mathrm{stage}2,\;\mathrm{currenct}\;\mathrm{epoch}\;>p_{set} \end{array}\right.$$

where $\lambda_{1}$ and $\lambda_{2}$ are weighting coefficients.

## 4. Experiments and Results

### 4.1. Datasets

To validate the effectiveness of the proposed LPC-Net model, two datasets were employed. The Carbon Fabric Defect Dataset (CF Defect Dataset), a self-built dataset, was used to assess segmentation performance on single-class defects. Meanwhile, the Magnetic Tile Defect Dataset (MT Defect Dataset) [1], a public dataset, was adopted to evaluate performance on multi-class defect segmentation.

**Figure 9:**
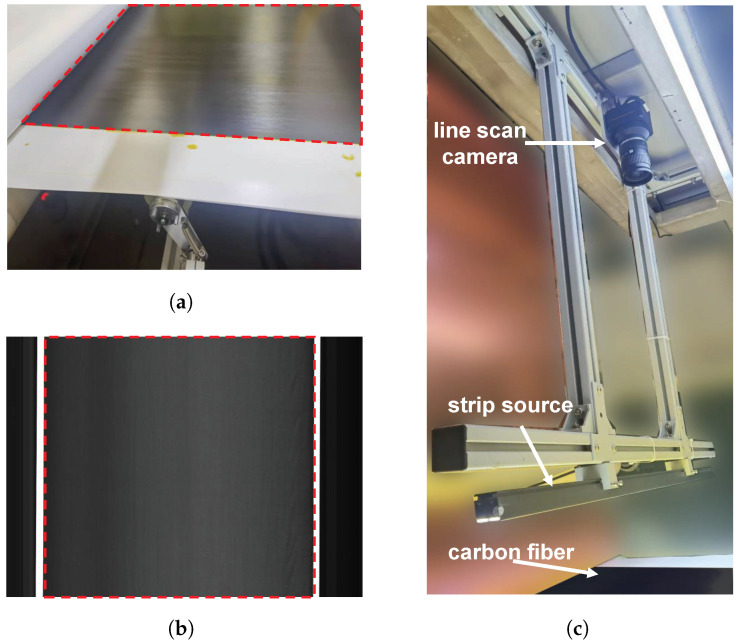
CF Defect Dataset industrial conditions. (**a**) Carbon fabrics. (**b**) Imaging effects. (**c**) Equipment setup for collection.

The CF Defect Dataset was collected from a real-world carbon fiber fabric production line using industrial line-scan cameras. As shown in Figure 9, the dataset acquisition setup, fabric samples, and imaging results are illustrated, with the red rectangular box indicating the target inspection region. The dataset consists of 233 samples with hairball defects collected from different production batches and coating conditions. Each image has a resolution of 512×512 pixels and is downsampled to 256×256 before being fed into the model. The samples exhibit significant variations in size, edge contours, and orientation, which pose challenges for conventional machine vision methods.

To further evaluate the model’s performance on multi-class defects, the MT Defect Dataset [1] was selected. This dataset contains 392 samples with varied resolutions and corresponding ground-truth labels. It includes five defect types: blowhole, crack, fray, break, and grinding uneven. All samples were resized to 256×256 pixels for consistency. Compared to the CF Defect Dataset, the MT Defect Dataset has a wider range of defect types and presents a greater challenge in segmentation.

Both datasets were randomly split into training, validation, and testing sets at a ratio of 3:1:1. For defect types with fewer samples, manual adjustments were made during the splitting process to ensure that all defect categories were included in the training, validation, and testing sets. This approach prevents class omissions and ensures a representative performance evaluation.

### 4.2. Performance Metrics

The network outputs binary segmentation maps, where black regions represent the background and white regions denote defect areas. To evaluate the model’s pixel-level segmentation performance while adhering to the industrial tolerance threshold of a maximum 20% error in defect area estimation, three metrics are adopted: mPA, mIoU, and $mIoU_{80\%}$. Their definitions are as follows:

(4) $$mPA=\frac{1}{k}\sum_{i=0}^{k}\frac{p_{ii}}{\sum_{j=0}^{k}p_{ij}}$$

(5) $$mIoU=\frac{1}{k}\sum_{i=0}^{k}\frac{p_{ii}}{\sum_{j=0}^{k}p_{ij}+\sum_{j=0}^{k}p_{ji}-p_{ii}}$$

(6) $$mIoU_{80\%}=\frac{\sum_{s=0}^{n}k_{s}}{n},\;k_{s}=\left\{\begin{array}{cc} 1 & ,\;mIoU(s)\geq 0.8\\ 0 & ,\;mIoU(s)<0.8 \end{array}\right.$$

where *n* denotes the number of test samples, *k* denotes the number of categories, and $p_{ij}$ is the number of pixels belonging to class *i* but predicted as class *j*; $mIoU_{80\%}$ indicates the proportion of samples in the test set with an mIoU greater than 80%.

### 4.3. Experimental Setup

The entire model was implemented in PyTorch 1.12.1 using PyCharm 2022.1.4, and executed on an NVIDIA Tesla A100 GPU (40 GB) (Nvidia, Santa Clara, CA, USA) under the CentOS 8 Linux environment.

The hyperparameter configuration is as follows: $\lambda_{1}=0.8$, $\lambda_{2}=0.2$, ω=0.2, ε=0.2, and $p_{set}=250$. The batch size was set to 16. The model was trained using the Adam optimizer with a base learning rate of 0.001, momentum parameters $\beta_{1}=0.5$ and $\beta_{2}=0.999$, and weight decay of 0.0001. The total number of training epochs was 400, with 100 epochs for the first stage and 300 for the second stage.

### 4.4. Experiment and Analysis

To evaluate the effectiveness of the proposed method, experiments were conducted on both the CF Defect Dataset and the MT Defect Dataset, comparing them against baseline and SOTA methods. The performance metrics are summarized in Table 2. On the CF Defect Dataset, the LPC-Net model achieved improvements of 1.28%, 1.86%, and 4.44% in mPA, mIoU, and ${mIoU}_{80\%}$, respectively, compared to the baseline DeeplabV3+. On the MT Defect Dataset, LPC-Net outperformed the baseline by 3.93%, 1.16%, and 4.35% in mPA, mIoU, and ${mIoU}_{80\%}$. Compared with the average performance of other methods, LPC-Net achieved gains of 1.58%±0.80% and 2.66%±1.12% in mPA, 1.35%±0.77% and 1.44%±0.79% in mIoU, and 4.24%±1.52% and 4.28%±1.96% in ${mIoU}_{80\%}$ on the CF Defect Dataset and MT Defect Dataset, respectively.

Overall, all models performed better on the CF Defect Dataset than on the MT Defect Dataset. Moreover, compared to the Deeplabv3+ benchmark model, LPC-Net demonstrated greater performance on the MT Defect Dataset, likely due to the increased complexity of the MT Defect Dataset, which includes more diverse defect types and more severe foreground–background imbalance. The relatively poor performance of SOTA models on both datasets may be attributed to their reliance on pre-trained weights. These models struggle to capture the local spatial dependencies when fine gaps exist within defects, often misclassifying such regions as background. Additionally, the limited sample size of both datasets may constrain the effectiveness of fine-tuning pre-trained models.

**Figure 10:**
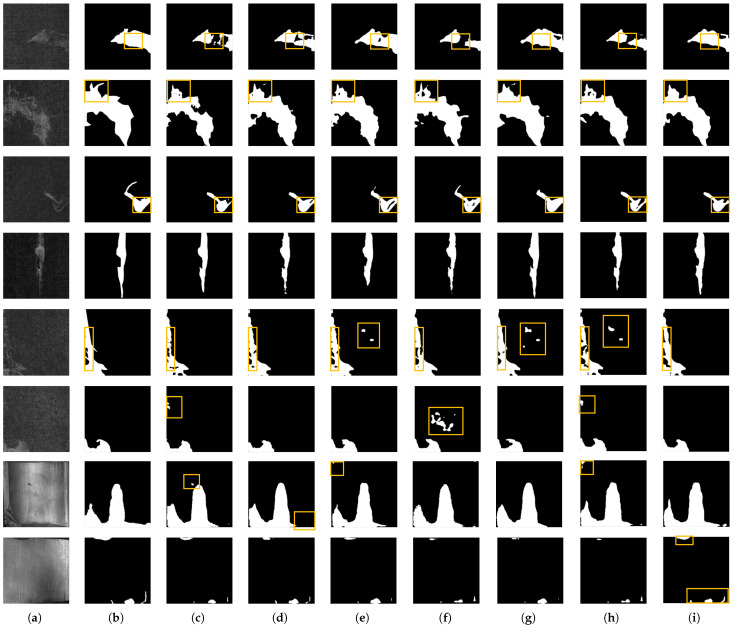
The performance of various methods on the CFD Datasets and the MT Defect Dataset. (**a**) Original image. (**b**) Ground truth labels. (**c**) FCN [19]. (**d**) U-Net [21]. (**e**) PIDNet [35]. (**f**) DDRNet [36]. (**g**) K-Net [37]. (**h**) DeepLabV3+ [34]. (**i**) LPC-Net.

Figure 10 presents the segmentation results of defect samples from the CF Defect Dataset and MT Defect Dataset using different methods. Column (a) shows the original images, column (b) provides the ground truth, and columns (c) to (i) display the segmentation results from various models. The yellow box shows the distinct differences in the segmentation results of different methods.

By comparing the first to third rows, it can be observed that when fine internal gaps exist within hairball defects, the proposed algorithm, under the guidance of the PCM, utilizes neighborhood probability distributions to reclassify these gaps as part of the defect region. The fourth row shows that when no internal gaps are present, the LPC-Net produces segmentation results comparable to other models. The fifth row illustrates that when large internal gaps within the defect should be identified as background, LPC-Net effectively distinguishes between the defect and background regions. Results in the second, fourth, and sixth rows further demonstrate that, under varying ratios of defects to background areas, the DLM enables the precise localization of defect regions, mitigating the performance degradation caused by foreground–background imbalance. The results in the seventh and eighth rows demonstrate that the network maintains robust segmentation performance across diverse defect types within the MT Defect Dataset, even when confronted with the simultaneous defects of different types in the eighth row. Additionally, segmentation results in column (i) show that the PCM in the LPC-Net reduces the steepness of segmentation boundaries at the defect’s edges, leading to smoother contours. This is due to the compensatory effect of the PCM on the areas with high boundary kurtosis, as discussed in Section 3.3.

Furthermore, as detailed in Table 3, we quantitatively evaluated the performance metrics of both model stages. The first stage is explicitly designed as a lightweight network, featuring only 0.16 million parameters, 0.65 GFLOPs, and achieving 794.2 FPS. This confirms its minimal computational footprint and fast processing speed. In contrast, the second stage prioritizes segmentation accuracy, consequently demanding greater computational resources. Nevertheless, it maintains a high throughput of 150.1 FPS and satisfies real-time operational requirements in industrial settings.

### 4.5. Ablative Study

To evaluate the effectiveness of the proposed DLM and PCM, as well as the influence of the weighting parameters $\lambda_{1}$ and $\lambda_{2}$ on segmentation performance, a series of ablation studies were conducted.

Ablation experiments were conducted by comparing the original Deeplabv3+ model, the LPC-Net model with only the DLM enabled, the LPC-Net model with only the PCM enabled, and the full LPC-Net model with both modules enabled. The results are presented in Table 4.

Compared to the original Deeplabv3+, enabling either the DLM or PCM led to noticeable improvements in mPA, mIoU, and ${mIoU}_{80\%}$ on the CF Defect Dataset. When both modules were enabled, the model achieved the highest performance across all three metrics. These results demonstrate that the DLM and PCM effectively enhance the segmentation capability of the model. On the MT Defect Dataset, the DLM alone also delivered significant gains in mPA, mIoU, and ${mIoU}_{80\%}$ over the baseline. However, the PCM’s impact was more limited when used independently, showing only marginal gains in mPA and a slight decrease in mIoU and ${mIoU}_{80\%}$. The limited efficacy of the PCM on the MT Defect Dataset stems primarily from the dataset’s characteristics: many defect types in the MT Defect Dataset lack the fine internal gaps or complex boundary ambiguities within defects that the PCM is specifically designed to resolve. Consequently, its corrective function offers less advantage here.

We also examined how model performance is affected by $\lambda_{1}$ and $\lambda_{2}$ values, corresponding to the weight of ${Loss}_{img}$ and ${Loss}_{pcm}$ on the CF Defect Dataset. The specific parameter settings and experimental results are shown in Table 5. As observed, moderately increasing the weight of ${Loss}_{pcm}$ improves segmentation accuracy. This is due to the PCM’s role in correcting internal gaps within defect regions. However, when the weight of ${Loss}_{pcm}$ is set too high, segmentation performance deteriorates. This is because the PCM smooths the gradient sharpness at the defect’s boundaries and excessive weighting leads to over-smoothing, causing a loss of boundary detail. Further experiments show that the model achieves optimal performance when the ratio of $\lambda_{1}$ to $\lambda_{2}$ is set to 0.8:0.2.

## 5. Conclusions

For industrial defect images characterized by a significant imbalance between defect and background areas or the presence of fine internal gaps within defects, conventional segmentation algorithms often suffer from foreground–background area imbalance and over-segmentation of fine gaps, which degrades overall performance. To address these issues, we propose a novel LPC-Net architecture, which integrates the DLM and PCM into the original DeepLabV3+ framework. The DLM utilizes rectangular bounding box labels as ground truth and employs a lightweight network to preliminarily locate defect regions. The resulting location feature vectors are fed into the second-stage main segmentation network, guiding it to focus on relevant defect areas. The PCM analyzes the neighborhood probability distributions of pixels in the initial segmentation results to generate a pixel confidence matrix. This matrix is then used to refine the initial predictions, reducing the over-segmentation of fine defect gaps. Experimental results demonstrate that, compared to the other network, the improved LPC-Net achieves gains of 1.58%±0.80%, 1.35%±0.77%, and 4.24%±1.52% in mPA, mIoU, and ${mIoU}_{80\%}$, respectively, on the CF Defect Dataset. On the MT Defect Dataset, LPC-Net improves mPA, mIoU, and ${mIoU}_{80\%}$ by 2.66%±1.12%, 1.44%±0.79%, and 4.28%±1.96%, respectively. These enhancements translate to more reliable automated quality assurance and reduced defect misjudgment costs in industrial production environments.

Future work will focus on two critical directions: First, while our ablation study validates the impact of manually tuned hyperparameters ($\lambda_{1}$ and $\lambda_{2}$) on performance, the sensitivity of deep learning models to hyperparameters necessitates further research. We will develop adaptive hyperparameter tuning strategies to dynamically balance multi-task loss weights, moving beyond static manual configuration. Second, the current method is designed for binary segmentation. To address the diversity of industrial defects, we will generalize the framework to support multi-class defect detection with task-specific annotation schemes, enabling the prioritized recognition of distinct defect categories.

## Figures and Tables

**Table 1:** The detailed parameters of different layers of backbone.

Layer Name	Output Size	Type
Input Image	1×256×256	/
Bottleneck block1	256×64×64	$\begin{array}{c} conv\;64,1\times 1\\ conv\;64,3\times 3\\ conv\;256,1\times 1\\ downsample=conv\;256,1\times 1 \end{array}$
Bottleneck block2	256×64×64	$\begin{array}{c} conv\;64,1\times 1\\ conv\;64,3\times 3\\ conv\;256,1\times 1\\ downsample=none \end{array}$
Bottleneck block3	512×32×32	$\begin{array}{c} conv\;128,1\times 1\\ conv\;128,3\times 3,stride=2\\ conv\;512,1\times 1\\ downsample=conv\;512,1\times 1,stride=2 \end{array}$
Bottleneck block4	512×32×32	$\begin{array}{c} conv\;128,1\times 1\\ conv\;128,3\times 3\\ conv\;512,1\times 1\\ downsample=none \end{array}$
Bottleneck block5	1024×16×16	$\begin{array}{c} conv\;256,1\times 1\\ conv\;256,3\times 3,stride=2\\ conv\;1024,1\times 1\\ downsample=conv\;1024,1\times 1,stride=2 \end{array}$
Bottleneck block6	1024×16×16	$\begin{array}{c} conv\;256,1\times 1\\ conv\;256,3\times 3\\ conv\;1024,1\times 1\\ downsample=none \end{array}$
Bottleneck block7	2048×16×16	$\begin{array}{c} conv\;512,1\times 1\\ conv\;512,3\times 3\\ conv\;2048,1\times 1\\ downsample=conv\;2048,1\times 1 \end{array}$
Bottleneck block8	2048×16×16	$\begin{array}{c} conv\;512,1\times 1\\ conv\;512,3\times 3\\ conv\;2048,1\times 1\\ downsample=none \end{array}$
Upsample1	256×64×64	upsampleby4,mode=bilinear
Upsample2	1×256×256	upsampleby4,mode=bilinear

**Table 2:** The segmentation indexes of the different methods.

	CF Defect Datasets			MT Defect Dataset		
Model	mPA(%)	mIoU(%)	${\mathrm{mIoU}}_{80\%}\left(\%\right)$	mPA(%)	mIoU(%)	${\mathrm{mIoU}}_{80\%}\left(\%\right)$
FCN	92.28	87.12	84.44	81.00	74.22	43.48
U-Net	93.37	88.79	86.67	79.17	75.06	44.93
PIDNet	91.68	88.07	86.67	82.22	76.16	47.83
DDRNet	92.96	88.90	88.89	81.99	75.59	46.83
K-Net	94.11	89.28	88.89	81.84	73.91	42.03
DeepLabV3+	93.24	87.82	86.67	79.72	75.32	44.93
LPC-Net	94.52	89.68	91.11	83.65	76.48	49.28

The values in the above table are the mean values of three repeated experiments.

**Table 3:** Computational efficiency of the two stages of the model.

Stage	Parameters	GFLOPs	Latency	FPS
First stage	0.16 M	0.65	1.26 ms	794.2
Second stage	39.75 M	14.84	6.66 ms	150.1
LPC-Net (total)	39.91 M	15.49	7.92 ms	126.2

**Table 4:** Ablation experiments of the DLM and PCM.

	CF Defect Dataset			MT Defect Dataset		
**Model**	mPA(%)	mIoU(%)	${\mathrm{mIoU}}_{80\%}\left(\%\right)$	mPA(%)	mIoU(%)	${\mathrm{mIoU}}_{80\%}\left(\%\right)$
deeplabv3+	93.24	87.82	86.67	79.72	75.32	44.93
LPC-Net (DLM)	94.22	89.40	88.89	83.30	76.88	47.83
LPC-Net (PCM)	94.00	89.60	86.67	80.20	75.22	43.48
LPC-Net (DLM + PCM)	94.52	89.68	91.11	83.65	76.48	49.28

**Table 5:** Ablation experiments with parameter settings of $\lambda_{1}$:$\lambda_{2}$.

$\lambda_{1}$:$\lambda_{2}$	mPA(%)	mIoU(%)	${\mathrm{mIoU}}_{80\%}\left(\%\right)$
1.0:0	93.24	87.82	86.67
0.9:0.1	93.64	88.15	86.67
0.85:0.15	94.08	88.96	88.89
0.8:0.2	94.00	89.60	86.67
0.75:0.25	93.08	88.03	84.44
0.7:0.3	92.27	88.60	86.67

## Data Availability

The data that support the findings of this study are available from the corresponding author upon reasonable request.
